# Home Department

**Published:** 1886-03

**Authors:** 


### HOME DEPARTMENT.

**Cranberry Sauce.**—Pour boiling water on your cranberries and let them simmer a few minutes, strain through a colander, then add sugar and boil ten minutes. No cranberry sauce is as fine as this. Gooseberries prepared the same way make a fine sauce.

**To Take Cinders From the Eye.**—In most cases a simple and effective cure may be found in one or two grains of flaxseed which can be placed in the eye without pain or injury. As they dissolve, a glutinous substance is formed, which envelopes any foreign body that may be under the lid, and the whole is easily washed out. A dozen of these seeds should constitute every traveller's outfit.

**Vegetable Soup.**—Peel and cut up very fine three onions, three turnips, one carrot and four potatoes, put them into a stew pan with a quarter of a pound of butter, the same of ham and a bunch of parsley; pass them two minutes over a sharp fire; then add a good spoonful of flour, mix well in, moisten with two quarts of broth, and one pint of boiling milk; boil up, stirring the while; season with salt and sugar, strain.

**To Take Fat off Soups, Gravies, &c.**—Thoroughly wet a cloth, such as a glass cloth, with cold water, and pour the stock through it; every particle of fat remains in the cloth, and your stock is as free from fat as if it had been allowed to get cold, and the fat removed in a cake. This hint will be found very useful, especially where beef tea, soup or jelly has to be prepared for invalids, which is often needed in a hurry. This fat can be melted and clarified, and is quite as good when removed from the cloth as if taken off in cakes.

**Chocolate Pudding.**—Grate two ounces of sweet chocolate; put it over the fire in a sauce-pan and melt it by gentle heat; heat a quart of milk quickly, stir it into the melted chocolate and let the mixture cool: separate the yolks and whites of six eggs, when the chocolate is nearly cool mix the yolks with it, add four tablespoonfuls of sugar, or more if required, and bake the pudding in an earthen dish; set in a pan of hot water for twenty minutes; meantime beat the six whites to a froth, add to them twelve heaping tablespoonfuls of powdered sugar, mixing the sugar very gently with the whites to form a meringue; put the meringue on top of the pudding and return it to the oven to color; then take the pudding from the oven and serve it either hot or cold.

**Baked Winter Squash**.—Winter squash may be cooked in various ways, and there is a considerable variety of them. The hard shell are the best for cooking. Wash them and break them in pieces; or, if the shell is soft enough, cut in two and remove the seeds; cut into pieces of convenient size and lay the shells downwards in a dish or bread pan; pour on a little boiling water to start with, place in a hot oven, and bake until soft. When done, the squash is dry and mealy.

**Cooking Potatoes.**—Pare the potato and slice it up, but not too thin. Place the slices in a large pie-dish, as if you were to make an apple-pie. Pour into the dish a very little water, drop a few slices of butter upon the potatoes, sprinkle them with salt and pepper, cover the whole with another plate, and set the dish in a hot oven. Twenty minutes' time is sufficient for the baking. The writer has tried this rule, and always with success. The potatoes have a distinctive flavor to be gained by no other method of cooking.

**Knub Celery**.—Wash the roots thoroughly, trim away the leaves and stalks and boil twenty minutes; drain, and when cool enough to handle, peel and cut into pieces of equal size; put these in a small baking-tin and add just enough warm milk to prevent drying up while cooking. Season with salt and pepper, and a walnut of butter; strew over the top a layer of crumbs, on top of which put a small pat of butter, and bake to a delicate brown. Some prefer a mixture of grated cheese and crumbs.

**Roast Chestnuts and Turkey**.—This is one of the latest "roasts" with which somebody has favored the cookery world, and as chestnuts are good and don't spoil the turkey, we give our readers the benefit of it: When the turkey is prepared, roast chestnuts enough to fill it. Remove the shells and the inner skins and fill the bird half full. Then add four ounces of butter and finish filling with the chestnuts. Sew up the bird, truss it and bake or roast it. Serve hot with the gravy only.

**Graham Crackers**.—Take sifted graham flour as for hard rolls, only a trifle stiffer. Use cold water—ice water is best—and knead thoroughly and very hard, fully twenty minutes; then roll to the thickness of ordinary pie-crust, cut in any shape desired and prick deeply with a fork. Bake from ten to fifteen minutes, or until the crackers are dry and hard. They should get entirely cold before they are put away, and then should be in a dry cool place, where no dampness can affect them. In using the ordinary graham flour, it will be an improvement to the crackers to add one-third or one-fourth part of white flour.

**Mashed Potatoes.**—To make mashed potatoes good and palatable pare them before boiling. Cook dry and mealy, then salt, mash and moisten with boiling milk and a piece of butter. Beat up light with a spoon or fork, put into a hot dish, and they are ready for the table. Any left over can, by adding one beaten egg, be made into round cakes and fried for breakfast, or may be made into croquettes, pear-shaped, and browned in the oven for the next dinner ; or it may be put into a vegetable dish, with a few bits of butter on the top, and browned in the oven.

**Chicken Salad.**—One large chicken boiled till tender : when cool take all the meat from the bones ; use it all except the skin ; cut it up in small pieces ; to a quart of chicken, add 1 pint of celery cut fine. Dressing : One tablespoonful of mustard, moistened ; piece of butter the size of an egg, ½ teaspoonful of salt, a little pepper, ½ cup of vinegar. Put all on the stove to scald. Beat 3 eggs and stir into the vinegar, not too hot, but hot enough to make it thick. Pour this over the prepared chicken. Add salt and pepper to taste.

**A Palatable Supper Dish.**—Line a vegetable dish with well-seasoned mashed potatoes ; leave a large space in the centre, wet it over with the white of an egg, or with milk sweetened with a very little sugar, and put it in the oven to brown delicately ; take about two dozen oysters, and a little milk, with butter, pepper and salt, and let it come to a boil on the top of the stove. Put in with the oysters a few thin slices of cold roast beef ; when this is sufficiently heated and the oysters cooked, pour it into the space left in the potato-lined dish.

**Milk as Food.**—"Go to the ant, thou sluggard, consider his ways and be wise," is an old injunction and a good one. We particularly commend it to those wise writers on the best forms of diet who condemn the use of milk for adults. If we should go to the ant, take a magnifying glass and watch his movements, we would find that the ant milks an insect bug and uses the milk, not for his babes, but for the adult ants. If the anti-milk-eating advocates will tell us when cows bags became distended so as to give more milk than their calves wanted, we should be happy to announce the time. We do not speak of the stuff called milk by the people of our great cities, but of genuine milk as it is given us by a good cow. There is no better food for children of all ages than good pure milk. We know of no exception to the rule. There may be stomachs that cannot bear it, but they are exceptions to the general rule.

### RECENT PUBLICATIONS.

**The Modern Crematist** is a new and novel candidate for public favor ; published at Lancaster, Pa. ; the first of its class in the United States. It is very neatly gotten up. Monthly, 15 pages, $1.00 a year.

**The Carrier Dove.**—This is a paper published in Oakland, Cal., and is one of ihe most sensible and able we know of under the name of Spiritualism, which it ably advocates and defends. It is a large quarto, and is filled with choice reading; has a number of portraits of persons eminent in Spiritualism, and, altogether, it is a magazine that should attract those who want to know about Spiritualism. It is published monthly at Oakland, Cal., at $2.00 per annum.

**The Century**, for March, is fully up to its great reputation. The continued description of the war, by different actors in the great tragedy, makes it of national importance. It is also a theme in which every American is and will ever be interested, no matter by whom told or how looked upon. The time has come when all can look back to the terrible war with coolness, and see how events crowded it along. From month to month these articles are looked for with great interest. The last number of the Century has a review of Shiloh, which, with the splendid maps accompanying, must interest every reader. Then, the fronticpiece of the present month is a most noticeable one, a splendid likeness of the greatest Spanish orator of the present age, and the great republican and admirer of the United States and its government—Castelar—a name that is still great in Spain, although he is known as a republican. The likeness alone is worth the cost of the whole volume. But we cannot designate. It is all good and should be in the hands of all who read.

---

**Mothers.**—There are upon the skin of every human being, child and adult alike, 2,300,000 pores. Through these pores in the form of insensible perspiration, is expelled more than one-quarter of the nourishment taken into the system. The importance of keeping open these perspiration valves upon a child's body, is second only to that of promptly digesting the food eaten. It was to open the pores of the skin and to assimilate the food that Dr. Pitcher formulated Castoria. Loose bowels, constipation, fevers and eruptions. which are so constant among infants and children, and which kill one-third of all children before they are five years old, arise principally from these two causes. It is from the wonderful results attendant upon the use of Castoria in regulating the stomachs and bowels, and keeping open the pores of the skin, that Castoria acquired its world wide reputation. With plenty of water for the body pure air for the lungs, and Castoria to assimilate the food, there need be no unaccounted for sickness among children. Castoria is a vegetable prescription without morphine or other narcotic property. Thirty years extensive use has given it a history never attained by another medicine.

